# Pollinator importance networks illustrate the crucial value of bees in a highly speciose plant community

**DOI:** 10.1038/s41598-017-08798-x

**Published:** 2017-08-21

**Authors:** Gavin Ballantyne, Katherine C. R. Baldock, Luke Rendell, P. G. Willmer

**Affiliations:** 1 University of St Andrews, School of Biological Sciences, St Andrews, KY16 9TH UK; 2 Edinburgh Napier University, School of Applied Sciences, Edinburgh, EH11 4BN UK; 3 University of Bristol, School of Biological Sciences, Bristol, BS8 1TQ UK; 4 University of Bristol, Cabot Institute, Bristol, BS8 1UJ UK

## Abstract

Accurate predictions of pollination service delivery require a comprehensive understanding of the interactions between plants and flower visitors. To improve measurements of pollinator performance underlying such predictions, we surveyed visitation frequency, pollinator effectiveness (pollen deposition ability) and pollinator importance (the product of visitation frequency and effectiveness) of flower visitors in a diverse Mediterranean flower meadow. With these data we constructed the largest pollinator importance network to date and compared it with the corresponding visitation network to estimate the specialisation of the community with greater precision. Visitation frequencies at the community level were positively correlated with the amount of pollen deposited during individual visits, though rarely correlated at lower taxonomic resolution. Bees had the highest levels of pollinator effectiveness, with *Apis*, *Andrena*, *Lasioglossum* and Osmiini bees being the most effective visitors to a number of plant species. Bomblyiid flies were the most effective non-bee flower visitors. Predictions of community specialisation (H_2_′) were higher in the pollinator importance network than the visitation network, mirroring previous studies. Our results increase confidence in existing measures of pollinator redundancy at the community level using visitation data, while also providing detailed information on interaction quality at the plant species level.

## Introduction

Biodiversity comprises both the species richness of a habitat and the interactions occurring between species. These interactions are even more vulnerable to change than the species themselves^1, 2^ and their loss may lead to failure of ecosystem function^3^. To understand how robust these interactions are to change and the likely outcome under altered conditions, it is essential that we attempt to measure the strength of those interactions; at the community scale, this may be challenging. For pollination biologists, understanding the quality of interactions between flower visitors and plants involves measuring the nature of the visits that take place to learn about resource rewards for visitors (e.g. ref. 4) and pollination services for plants (e.g. ref. 5) in the context of the community ecology. To improve the accuracy of pollinator performance estimates Ballantyne *et al*.^6^ constructed the first community interaction network that incorporates both visitation frequency to flowers and pollinator effectiveness (single visit pollen deposition onto stigmas) of those visitors into a more accurate measure, termed ‘pollinator importance’ (the potential importance of the visitor for the plant). Here we study pollinator importance in a Mediterranean flower meadow, with high plant and pollinator species richness, to test the limitations of such community-wide measurements and investigate visitor performance at the species and community levels (we hereafter use “performance” as a general term referring to likely pollination service provided, as measured at the point of the visit, sensu Ne’eman *et al*.^7^).

Assessing the value of flower visitors to plants involves analysing behaviour at different scales, from visitation frequency, to ability to pick up pollen (pollen export capacity), and to deposit viable pollen on conspecific stigmas (quality of pollen deposition). It has been suggested that, due to the high variance in visitation frequency, the number of visits a flower receives from a given pollinator provides the most informative measure of its performance (e.g. ref. 8). Indeed, Vázquez *et al*.^9^ found that interaction frequency was a good predictor of interaction strength as measured by pollen tube growth in four arid shrubland species. However, for many plant species there is also high variance in potential pollination success due to the amount of pollen deposited^6^ or the quality of that pollen deposited^10^. Conclusions regarding the pollination specialisation of a plant community may be greatly influenced by the data used and the focus of the researchers^11, 12^, so potential biases inherent in estimates of pollinator performance need to be investigated. To accurately determine the potential of data-gathering techniques, they must also be carried out with a wide variety of flower visitors and a diverse range of floral morphologies.

We previously measured individual visit pollinator effectiveness in terms of pollen deposition for all five flowering species present during summer in a low diversity heathland community in the UK^6^. Pollen-gathering visitors deposited significantly more pollen on stigmas than nectar-feeders and, as expected, legitimate visitors deposited significantly more pollen than nectar robbers. For the first time measures of potential pollinator importance were used to construct a network of community-level plant-pollinator interactions. The resulting pollinator importance network was more specialised than a network produced using visitation frequency alone.

Estimates of potential pollinator importance are likely to improve predictions of the contribution of visitor species to the pollination of individual plant species. This is due to improvements in those species level metrics in network analyses that represent specific visitor species’ contributions to the pollination of the whole plant community. With such information we can improve predictions of the outcome of species change within flowering plant communities that could prove valuable in conservation^13^ as experimental removal of key pollinators from local communities may negatively impact plant fitness^14^.

Here we construct the first pollinator importance networks for a highly diverse and bee-rich floral community and address the following questions:

1. How well does visitation frequency predict pollinator effectiveness in a diverse plant community? A strong correlation between the measures in such a large dataset could support the general use of visitation frequency as a proxy for pollinator performance.
2. Which visitor groups are the most effective potential pollinators? Bees are likely to be the most effective for the majority of flower types^5^, but floral morphology will play a role in dictating which bees are best for particular plant species, due to matches of corolla shape and size with visitor body size and tongue length.
3. How do pollinator importance networks compare with visitation networks in a highly diverse habitat? We expect interactions in pollinator importance networks to be more specialised (H2′) and to show lower nestedness (weighted NODF, a metric describing link patterns at the community level, which may inform about network dynamics and breakdown patterns^15^), compared to visitation networks.
4. How do estimates of species level metrics, such as specialisation and contribution of service (pollen deposition or provision of resources) to the other trophic level, relate to plant and visitor morphological traits? Extremes of flower and visitor morphology, such as stigma accessibility or tongue length, are expected to correlate positively with high species-level specialisation^16^.

## Results

### Floral visitation and pollinator effectiveness

We observed 2270 insect visits to 23 plant species from 14 families over the study period (plant species in Table 1, visitor species identified in Supplementary Table S1). These comprised 179 unique interactions between plant species and visitor groups, 149 of which resulted in pollen deposition (Supplementary Table S3a).

**Table 1:** Plant study species, classified according to flowering season (early = early Feb to mid-March, late = mid-March to late April), nights bagged before observation (*Moraea sisyrinchium opens at midday and so bagging was unnecessary), stigma and reward accessibility (restricted access = 1, open access = 5), stigma and pollen size (small = 1, large = 5) and sample size.

Species	Family	Flowering Season	Nights Bagged	Stigma Accessibility	Stigma Size	Pollen Size	Flowers sampled
*Allium trifoliatum*	Liliaceae	late	1	4	2	5	73
*Asphodelus aestivus*	Xanthorrhoeaceae	early	1	5	3	5	98
*Bellevalia flexuosa*	Asparagaceae	early	2	3	2	5	79
*Centaurea cyanoides*	Asteraceae	late	2	5	2	4	104
*Cistus incanus*	Cistaceae	both	1	5	5	4	112
*Cistus salvifolius*	Cistaceae	late	1	5	5	5	100
*Convolvulus coelesyriacus*	Convolvulaceae	late	1	4	4	5	97
*Cynoglossum creticum*	Boraginaceae	both	2	2	1	1	86
*Echium judaeum*	Boraginaceae	late	1	4	2	2	140
*Hirschfeldia incana*	Brassicaceae	both	1	4	3	2	105
*Linum pubescens*	Linaceae	both	1	3	4	5	68
*Lomelosia prolifera*	Caprifoliaceae	late	2	4	4	5	119
*Moraea sisyrinchium*	Iridaceae	early	NA*	4	4	5	101
*Nonea obtusifolia*	Boraginaceae	early	1	2	2	2	70
*Ochthodium aegyptiacum*	Brassicaceae	both	1	4	2	2	79
*Ornithogalum narbonense*	Asparagaceae	early	1	5	3	5	78
*Phlomis viscosa*	Lamiaceae	late	2	1	5	4	105
*Prasium majus*	Lamiaceae	both	3	2	2	3	102
*Ruta chalepensis*	Rutaceae	late	3	5	3	3	92
*Salvia fruticosa*	Lamiaceae	early	3	2	2	3	161
*Scandix verna*	Apiaceae	early	2	5	1	3	101
*Stachys neurocalycina*	Lamiaceae	late	2	2	2	2	91
*Tordylium carmeli*	Apiaceae	late	2	5	1	3	109

The most frequent visitor group to individual plant species was the most effective at depositing pollen (PE) in only 5 of the 23 species (*Echium judaeum*, *Linum pubescens*, *Prasium majus*, *Ruta chalepensis* and *Salvia fruticosa*). However, the most frequent visitor group had the highest pollinator importance (PI) in 16 species, (perhaps unsurprising since PI also incorporates visit frequency). Visitation frequency was, as predicted, positively correlated with PE for all visitor categories combined (rho = 0.389, p < 0.001). However, for individual visitor groups frequency was only significantly, or marginally significantly, positively correlated with pollinator effectiveness for *Andrena* (rho = 0.496, p = 0.051) and syrphids (rho = 0.783, p < 0.005), although syrphids were relatively infrequent visitors and deposited less pollen in comparison with bees or bombyliids (see Supplementary Table S4).

### Pollinator effectiveness of visitor groups to individual plant species

**Figure 1:**
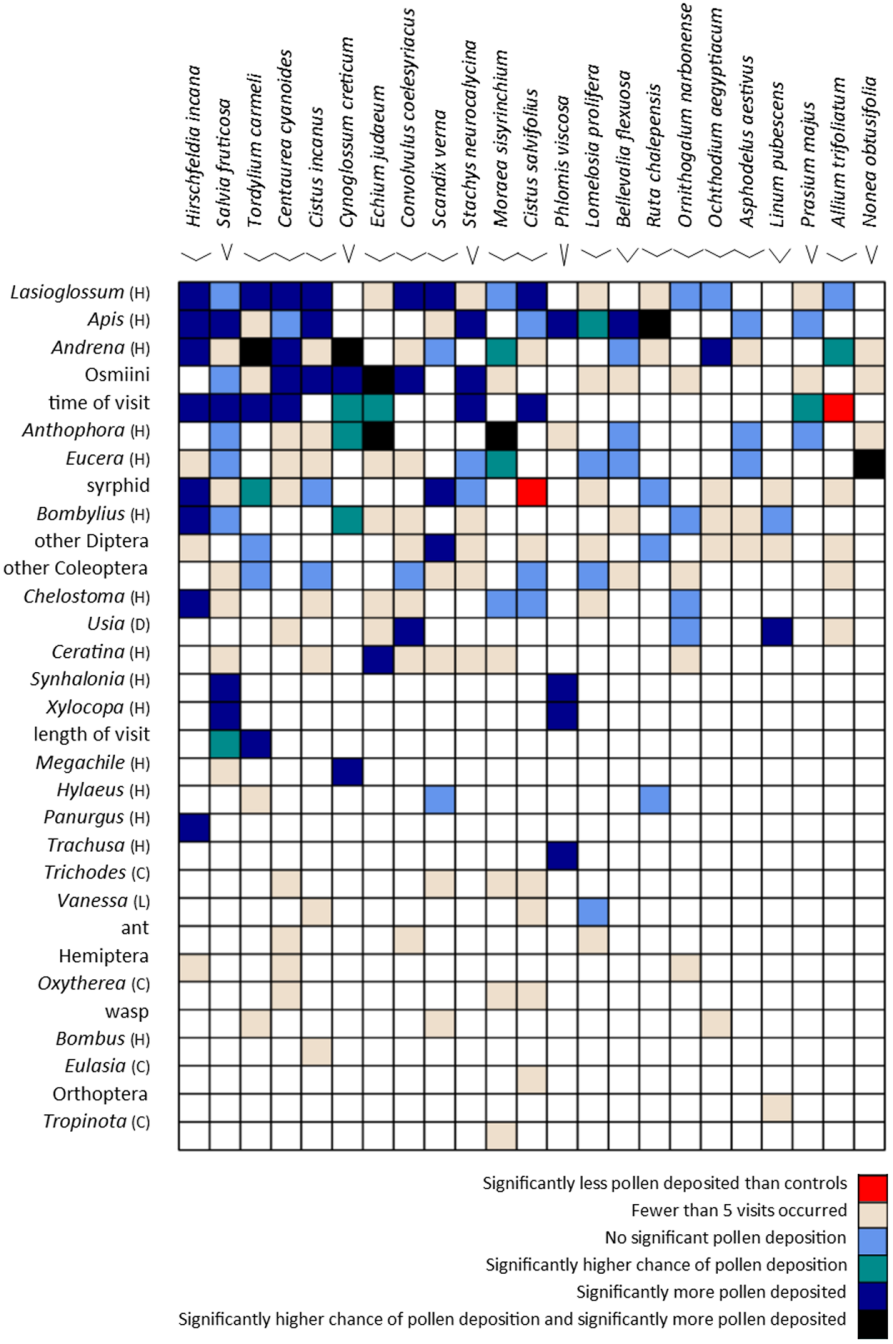
Community interaction matrix of visits to target plant species by visitor groups and the influence of covariates time of day and length of visit. Unique interactions are coloured to represent the following criteria from the hurdle model: fewer than five visits recorded and so not used in the analysis; no significant pollen deposition relative to controls, significantly higher likelihood of pollen deposition relative to controls; when pollen deposition occurs there is significantly more than controls; both of the latter categories. Significance was recorded at the p < 0.05 level. Stigma access is indicated by the accessibility of the inner joint of the line diagram by each plant name. Visitor genera identified by family: Coleoptera (C), Diptera (D), Hymenoptera (H) and Lepidoptera (L).

Hurdle models of pollen deposition by visitor groups to individual plant species that took into account the influence of time of day and length of visit (Fig. 1; Supplementary Table S5) showed that pollen-collecting bees were the most effective visitors to plant species in the community (predicted pollen counts were a good match for observed pollen deposition, Supplementary Fig. S1). *Lasioglossum* bees (the majority being *Lasioglossum marginatum*) deposited significantly more pollen than control levels onto seven out of the twelve species they visited frequently, all of which were species with easily accessible stigmas and floral resources. *Apis*, *Andrena* and Osmiini bees were regularly effective visitors both to species with easily accessible and harder to access floral rewards, with *Apis* being significantly effective visitors to 8 out of 12 frequently-visited plant species, *Andrena* to 7 out of 9 and Osmiini to 6 out of 7. *Anthophora* bees were not as effective for the species they visited, only contributing effective visits to 3 out of 7 species; this might be due to the high proportion of males observed earlier in the season, visiting very quickly and only for nectar.

The most effective non-bee visitors were bombyliid and syrphid flies, which deposited significant amounts of pollen during visits to some plant species. Syrphids, however, were common visitors but rather poor pollinators for *Cistus salvifolius*, as they deposited significantly less pollen than found on control flowers, possibly due to their habit of eating pollen already deposited on the tops of the large accessible stigmas of this species.

The specialised bees *Xylocopa*, *Synhalonia* and *Trachusa* were less important at the community level as they visited a small number of plant species, although they did deposit significant amounts of pollen on these. The only bees that were never effective flower visitors were small, smooth-bodied *Hylaeus* species (Colletidae) that collect pollen in their internal crop. Beetles, other flies and *Vanessa* butterflies also never deposited significant amounts of pollen.

**Figure 2:**
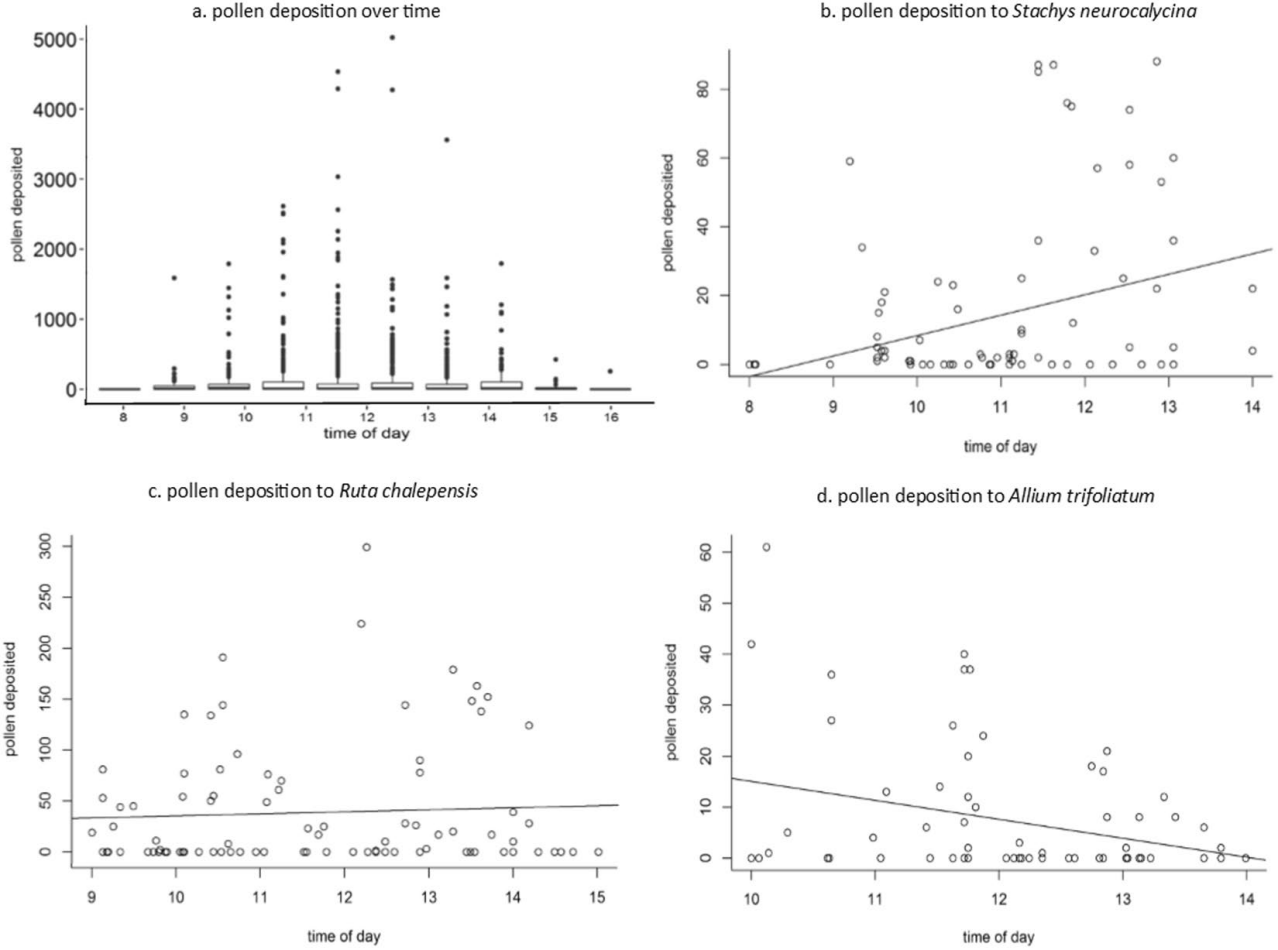
Diurnal changes in pollen deposition. (**a**) Pollen deposition shows no linear relationship with time of day at the community level (mean and standard errors per hour shown), but in the case of individual species, more pollen may be deposited as the day progresses (e.g. (**b**) *Stachys neurocalycina*; rho = 0.267, p = 0.011), show no relationship (e.g. (**c**) *Ruta chalepensis*; rho = 0.005, p = 0.962) or decline as the morning ends (e.g. (**d**). *Allium trifoliatum*; rho = −0.226, p = 0.054).

At the community level pollen deposition showed no relationship with time of day (rho = 0.004, p = 0.866), but did peak around 10:30, coinciding with the peak of pollen presentation (Fig. 2a). The hurdle models showed that significantly more pollen was deposited on stigmas as the day progressed for *Hirschfeldia incana, Salvia fruticosa, Tordylium carmeli, Centaurea cyanoides, Stachys neurocalycina* and *Cistus salvifolius*, while significantly less pollen was deposited on *Allium trifoliatum* later in the day as anthers that dehisced in the morning were quickly depleted of pollen (Fig. 2; Supplementary Table S5). Length of visit was only a significant predictor of pollen deposition for the labiate *Salvia* fruticosa and the umbellifer *Tordylium carmeli*.

### Community-level network analysis

All networks were more specialised than null model predictions (with significantly higher values of H_2_′). Both V and PI networks were less nested than null model predictions (with significantly lower values of weighted NODF). Observed numbers of interaction partners were also lower than model predictions (significantly lower plant and visitor generality) and interactions were less connected and less uniformly distributed (significantly lower connectance and evenness). Full details of null model predictions and z-scores can be found in Supplementary Table S6.

**Figure 3:**
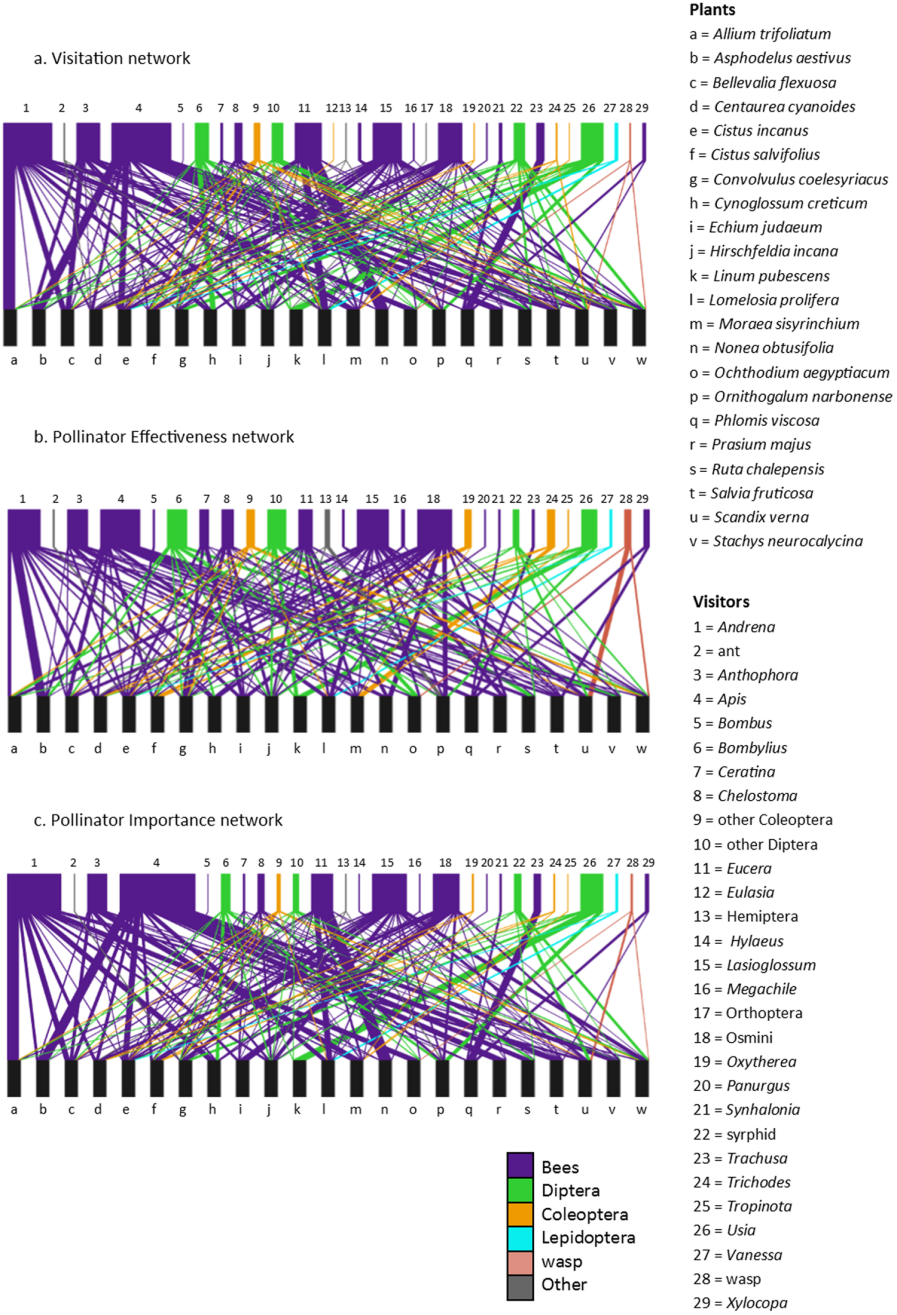
Visitation, Pollinator Effectiveness and Pollinator Importance interaction networks for sampled plant species on Mount Carmel.

Network level metrics (Table 2) show that the visitation (V) network interactions were moderately specialised, falling in the middle of the range with an H_2_′ of 0.55 (and an identical null model corrected value), and a relatively low level of nestedness of 24.6% (corrected value −18.65). As predicted, the interactions in the PI network were more specialised (H_2_′ = 0.62; corrected value 0.61). However, while nestedness was slightly lower in the PI network (21.6%), after correction with null model values it is, in fact, slightly more nested (corrected weighted NODF value −17.38) than the V network. Visitor and plant generality were also lower in the PI network compared to the visitation network, since interactions where no pollen is deposited are excluded from the network (Table 2). There was little difference in link evenness between the two networks, with a similar range of weak and strong links (Table 2, Fig. 3). The PE network had the lowest H_2_′ value (0.51; corrected value 0.50) and the highest corrected nestedness (7.07, which was not significantly different from null model predictions).

**Table 2:** Community level network metrics for the standard dataset, showing observed network metrics, mean and standard deviation of null model predictions (1000 null models) and metrics corrected by null model predictions. Number of interactions are controlled for in the null model, so are not predicted. Observed values in bold are significantly different from null model predictions (p < 0.001).

		V	PE	PI
No. interactions		2270		
No. links		179		
H′_2_	Observed value	**0.55**	**0.51**	**0.62**
	Null mean	0.005 ± 0.0003	0.005 ± 0.0003	0.006 ± 0.0003
	corrected value	0.55	0.50	0.61
Weighted NODF	Observed	**24.59**	16.45	**21.58**
	Null mean	43.24 ± 2.49	9.39 ± 5.39	38.96 ± 2.72
	corrected value	−18.65	7.07	−17.38
Evenness	Observed value	0.68	0.73	0.66
	Null mean	0.88 ± 0.0001	0.93 ± 0.0001	0.86 ± 0.0001
	corrected value	−0.20	−0.20	−0.20
Connectance	Observed value	**0.28**	**0.25**	**0.25**
	Null mean	0.90 ± 0.006	0.99 ± 0.002	0.92 ± 0.006
	corrected value	−0.61	−0.75	−0.68
Visitor generality	Observed value	**7.13**	**7.53**	**6.99**
	Null mean	22.71 ± 0.02	22.72 ± 0.02	22.73 ± 0.02
	corrected value	−15.58	−15.19	−15.73
Plant generality	Observed value	**3.97**	**5.18**	**3.33**
	Null mean	13.00 ± 0.01	17.11 ± 0.01	11.00 ± 0.01
	corrected value	−9.03	−11.93	−7.67

.

Comparisons of network level metrics across the network types were consistent across the different datasets; H_2_′ was consistently higher in the PI network when (i) small sample sizes where visitor groups contributed fewer than 5 visits to one plant species were removed from the dataset, (ii) visitor groups were reclassified to a more precise taxonomic resolution (using pollen deposition data shown in Supplementary Table S3b) and (iii) the network was divided into early and late season plant species (Table 2), despite the composition of visitor groups changing over the course of the season, with *Eucera* bees, for example, playing a greater role early in the season, and various Osmiini being more important later in the season. However, the exact effectiveness values for different visitors in the case of (ii) and (iii) should be treated with some caution as the splitting of the data results in some low sample sizes.

When only visitor groups that deposit significantly more pollen onto flowers compared to controls were included in PE and PI networks (a stricter definition of what might be considered an effective pollinator), both were more specialised than the visitation network (corrected H_2_′ = 0.77 and 0.81 respectively).

### Species/group-level metrics and morphological traits

**Figure 4:**
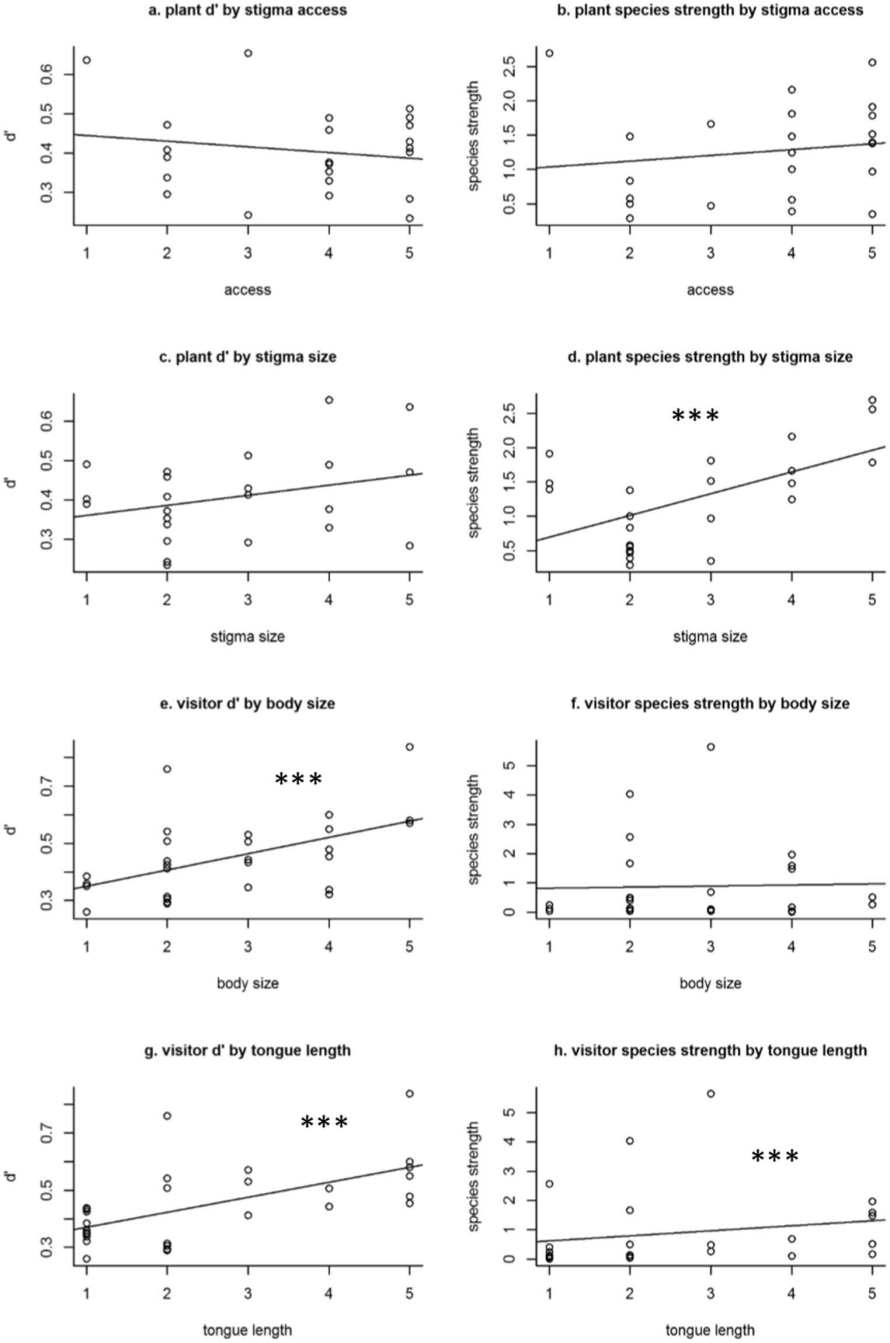
Correlations of species level network metrics d′ and species strength with plant traits (stigma accessibility, stigma size and pollen size) and with visitor traits (body size and tongue length). Significant positive correlation indicated by “***”.

Several morphological traits were correlated with species and group-level metrics (Fig. 4; species-level metrics Supplementary Table S7). Visitor tongue length was significantly correlated with d′ (rho = 0.637, p < 0.001; Fig. 4g), suggesting that visitor groups with shorter tongues were more generalised in their flower preferences. Tongue length was weakly correlated with species strength (rho = 0.497, p = 0.009; Fig. 4h). As larger visitors typically have longer tongues, a significant positive relationship also occurred between visitor body size and d′ (rho = 0.536, p = 0.003; Fig. 4f), but body size was not itself correlated with species strength (rho = 0.08, p = 0.7; Fig. 4f). Stigma size was positively related to species strength (rho = 0.479, p = 0.021; Fig. 4d), possibly because visitors to larger flowers with larger stigmas were more dependent on these plant species for resources than visitors to smaller flowers which tended to visit a wider range of species. No significant relationships were found between the other variables shown in Fig. 4, or between pollen size and any network metric, or between weighted closeness centrality and any plant or visitor trait.

## Discussion

### Measures of pollinator performance

These first pollinator importance data from a highly diverse plant and insect community confirm the prediction that interactions are more specialised when link weightings take pollen deposition into account. This change may be small at the community level, for reasons discussed below. However, our analysis of visitors’ pollen deposition ability to a wide range of plant species shows that pollinator importance data provide valuable information on individual species interactions in terms of pollen deposition ability and species level metrics of plant specialisation and visitor contribution. Our data also confirm that visitation frequency may act as a reasonable proxy for pollinator importance at the community level (building on the findings of Vázquez *et al*.^9^), but remains a poor predictor for pollen deposition ability for most visitor groups. We also demonstrate that collection of data on potential pollinator importance is feasible for interactions with common plants within a diverse community.

Both measures of pollinator performance (visitation and pollen deposition) were heavily dominated by bee species, as would be expected in a Mediterranean floral meadow with high densities of these pollen collecting insects. Small *Lasioglossum* bees had higher pollinator importance scores than all other visitors when visiting open flowers with accessible rewards, with *Apis*, *Andrena* and Osmiini also important visitors for many plant species of varying floral morphologies. The large specialist *Xylocopa* and *Synhalonia* bees performed extremely well at the few plant species that they visited.

Most non-bee groups appeared to be relatively ineffective pollinators for the plants we sampled, with low rates of both visitation and pollen deposition (cf ref. 17). The only exceptions were bombyliid flies, which our data suggest were important pollinators for several plant species. *Usia* spp. in particular are specialised pollinators of *Linum pubescens* ^18^ and this was reflected in both visitation frequency and pollen deposition for this plant. Despite having an almost identical floral structure and phenology to the more broadly attractive *Convolvulus coelesyriacus*, *L. pubescens* was almost totally avoided by pollen-collecting bees, indicating potential scent and/or nutritional specialisation through the use of secondary compounds. Syrphids were generally ineffective at depositing pollen on any plant species, but most syrphid visitors recorded were from small- or medium-sized species, and larger eristaline syrphids may be more effective when present in greater numbers as they are certainly effective at depositing pollen onto other plant species^19, 20^. Medium sized syrphids, especially *Episyrphus balteatus*, are also known to be effective pollinators in other situations (e.g. ref. 21).

Coleoptera had both low visitation frequency and low pollen deposition scores, resulting in very poor pollinator importance. They may however, be over-represented in traditional floral transect surveys (Ballantyne, unpublished data) as they spend long periods on individual flowers, rarely move between flowers, and are also less likely to be disturbed by a moving observer than other floral visitors such as hoverflies or hawkmoths, making them more likely to be counted in such surveys.

Visitation frequencies were positively correlated with pollen deposition in our study, supporting the general findings of Vázquez *et al*.^8^ that visitation can act as a surrogate for pollinator performance. This was mainly due to the dominance of bees at this site, which were frequent visitors and deposited large quantities of pollen, resulting in high pollination importance scores, as would be expected for visitors that actively collect pollen and whose face hairiness is positively correlated with pollen deposition^22^. While this correlation between visitation and pollen deposition was found at the community level, it rarely applied to individual visitor groups. Some frequent visitors were poor pollen depositors as they only collected nectar from the flowers and/or were poor morphological matches with the flowers (e.g. male *Anthophora* bees visiting *Asphodelus aestivus* or Diptera visiting *Tordilium carmeli*), a situation detected in numerous studies of single plant species (e.g. refs 23 and 24). Such visits will only influence plant fitness if the removal of floral rewards influences the behaviour of more effective pollinators. Others have also cautioned against relying solely on interaction frequencies to estimate the structure of plant-pollinator interactions^25–27^, as high numbers of low quality visits need not necessarily translate into effective pollination (e.g. refs 28 and 29). In the case of *Luffa* gourd, for example, the best predictor of seed set was not visitation frequency, but instead functional bee diversity in terms of pollen deposition^30^. As a consequence, visitation frequency should still be treated as a general indicator but not as a surrogate for pollinator importance. The likely pollinator predicted from floral pollination syndrome will usually be the most effective; but unexpected visitors may in fact be the most important, such as *Usia* to *L. pubescens* as here, insectivorous bats to *Pachycereus pringlei* ^31^, or infrequent visits by long tongued bumblebees to *Ipomopsis aggregata* ^32^.

### Community-level interactions

The increase in network specialisation when a visitation network is compared with the corresponding pollinator importance network, although small, is consistent with results from a low diversity, less specialised heathland plant community^6^. The moderately high specialisation observed in our results is reasonable given the diverse range of floral morphologies sampled. Although many species chosen for logistical reasons were highly generalised (as high visitation rates were needed for data collection to be practical), e.g. *Hirschfeldia incana*, others with equally high visitation frequency were specialised, e.g. *Phlomis viscosa* which is reliant on large bees. The entire community may be more specialised when all flowering plant species in the habitat are considered, as the increase in the diversity of functional floral traits should increase specialisation and lower the connectance^33^. Interactions recorded were also generalised by the need to aggregate data from across two flowering seasons and from throughout the day into the one network for analysis, a common necessity for time-consuming community studies (albeit with some notable exceptions^34, 35^). Nevertheless, when we considered our data as two separate seasonal time-points (early and late season) pollinator importance networks were still slightly more specialised than the corresponding visitation networks.

Our results support the widespread finding, and specifically the data from Chamberlain *et al*.^16^, that larger flower visitors (often with longer tongues) are more specialised, due to their habit of seeking out flowers with longer corollas which are more likely to be rewarding than those with shorter corollas that attract a wider range of visitors. However, while some broad patterns can be detected between morphological traits and species-level network metrics, metrics are often not correlated with individual traits.

### Methodological limitations and future steps

While providing more reliable information on plant pollination success than visitation frequency data, the biggest limitation of pollinator importance scores as used in this study remains the lack of information on the quality of the pollen deposited, and in particular on the viability of conspecific grains. Pollen quality (both the viability and the heterospecific content of the deposited grains, as well as the proportion of geitonogamous pollen for self-incompatible species) depends on various factors, including visitor dispersal distances and floral constancy, and can have a serious impact on seed set (e.g. refs 10 and 36). Pollen is likely to be dispersed further by larger bees^37^ and trap-lining species will disperse pollen further afield than those with strong floral constancy within patches, such as *Apis mellifera* ^5^. The amount of pollen needed for successful pollination will also vary between plant species^25^. As a consequence, pollinator importance should best be thought of as a measure of potential pollinator performance.

High levels of visitation may become damaging due to pollen saturation (stigma clogging), pollen removal from stigmas, tissue damage caused by visits, or nectar removal/contamination (e.g. refs 38 and 39). So, for a small number of plant species, those visitors with the highest values for pollinator effectiveness in networks may in fact be detrimental to particular flowers. However, this is unlikely to be an issue for most species as seed set usually plateaus (rather than ultimately declining) with increasing visitation frequency^5^.

The methodology used also imposes restrictions on ability to accurately estimate network metrics such as H_2_′. Not all flower-visitor interactions within the community could be measured, so results provide estimates of community structure skewed towad the most frequent interactions. Thus, the observed values will underestimate the true complexity of interactions at the site. Care should be taken when making comparisons with studies that do comprehensively sample rarer interactions. How we define an effective pollinator for a plant species also has a huge effect on the resulting network metrics. If only visitors that reliably deposit significantly more pollen than controls are considered, the PI network is much more specialised than the V network. However, while large quantities of pollen may be necessary for successful pollination for many plant species, smaller amounts of good quality pollen may be sufficient for others (e.g. ref. 40). Instead we focussed the majority of our analyses on networks that included the more conservative use of pollen deposition controls, subtracting the average control value of that plant species, so as not to exclude visitors that may often perform poorly, but that may occasionally deposit useful amounts of viable pollen. This gave a PI network that was only slightly more specialised than the V network, but as some of the pollen deposited may have been self-pollen moved by the visitor, the real degree of specialisation probably lies somewhere between the two estimates.

Estimates from networks constructed using data compiled from multiple phenological periods (such as ours), or from multiple geographical areas, may also drastically overestimate network robustness due to stacking of temporal data across plant flowering and visitor activity phenologies. When only those interactions occurring at any one time and place are examined, interaction links are likely to be more vulnerable^36, 41, 42^.

The correlation between visitor tongue length and specialisation (d′) should also be treated with caution, as it is potentially an artefact given that bigger more specialised visitor groups are more accurately identified to species from observation alone. The large bees with long tongues *Synhalonia* and *Trachusa*, for example, represent the single species *S. plumigera* and *T. pubescens* respectively, while lumped taxa, containing multiple species with similar shorter tongues, received less weight. While we recognise that the individual species and groups are not strictly independent due to shared evolutionary history, at the genus-level insect groups vary greatly in the range of plant species they visit^25^.

Despite these limitations, pollinator importance metrics identify when and where visitation frequency is a reliable guideline for visit quality and provide additional details about specific interactions. It will also be interesting to see how pollinator importance compares with pollen transport, which can be constructed using the product of pollen load and visitation frequency^43^. The two measures will complement each other, by identifying when visitors carry many pollen grains but deposit few, and when visitors carry very little pollen but deposit substantial self-pollen^20^.

## Conclusions

Our study shows that it is feasible to collect data showing potential pollinator importance for a substantial component of a complex and diverse flowering community. These data show that, in a diverse habitat where visitation is dominated by highly effective bee species, recording visitation is a broadly reliable substitute for pollen deposition data at the community level. Pollinator importance data also provides valuable information about individual species interactions as well as more accurate estimates of community specialisation. Careful incorporation of pollen deposition data into community-level studies will enhance our understanding of pollination services.

## Methods

### Study site and study species

Data were collected from early February to late April in 2014 and 2015 in a 0.35 km^2^ area of sheep-grazed, Mediterranean garigue scrub habitat on Mount Carmel National Reserve, Israel (32°43′48″N, 35°0′39″E). Plant species were selected if they were: (i) common enough that multiple patches of 50+ flowers could be found in multiple areas within the site, (ii) had a high enough visitation frequency to allow efficient data collection (at least 1 visit to any of 10 flowers within 30 minutes), (iii) did not have high levels of self-pollen contamination when measured on unvisited flowers, and (iv) contributed towards a morphologically and taxonomically diverse dataset (i.e. if two Brassicaceae had already been surveyed, a common species from an un-surveyed family would be selected for sampling over a third common Brassicaceae). This resulted in 23 study species, representing 35% of the plant species richness at the site during the survey period. Although sampling did not represent the entire plant community, our study allows for a valid comparison of techniques between the most frequently visited common species.

### Pollen deposition

We used single visit pollen deposition (SVD) data as our measure of pollinator effectiveness^6, 44^. Flower buds (or inflorescences containing buds) were bagged using mesh cages each evening throughout the study period. Bagged flowers only represented a small number of potential flowers available for visits. The cages were carefully removed the following day once flowers had opened, and receptive stigmas identified on flowers that had opened overnight using a hand-lens. In most cases this occurred on the first day following bagging, but a few species (usually those that were strongly protandrous) were unbagged two or three days later, depending on the time required for the stigma to become receptive (see Table 1). Such bagging may lead to greater rewards being present in observed flowers than the surrounding flowers, but the change in rewards remains consistent for all visitors.

Individual unbagged flowers were observed until they received their first visit, and the visitor identity and visit duration were recorded. Immediately after, stigmas were removed from flowers using fine forceps, and pollen from the surface of the stigma transferred onto a cube of fuchsin gel, which was melted and preserved on a slide, allowing pollen to be identified using a reference collection and counted under a compound microscope. Pollen could be identified to species in most cases, with the exception of *Cistus* spp. or Brassicaceae pollen, which had to be treated as conspecific pollen on plants of these taxa.

Surveys continued on each day until there were no more bagged flowers to sample, and/or visitation frequency had decreased to a very low level (no visits for 1 h). All data were collected between 07:00 and 17:00 h in warm dry conditions of 14 °C or above, with winds lower than 7 km/h. Data collection occurred at least 5 days a week throughout the study periods (approx. 1500 person hours), with between 68 and 161 flowers sampled per species (Table 1).

To account for pollen found on stigmas due to opening of the flower and/or handling and bagging procedures, control stigmas (11–20 per species) which had been bagged for the same time as observed flowers were also sampled, from newly unbagged flowers before any visit took place, and checked for pollen as above. Mean control values for each species were then subtracted from the SVD values for individual visits.

### Insect identification

Insect flower visitors were identified to species or genus by eye, or otherwise photographed and/or caught for later identification. To acquire realistic pollen deposition values visitors were always allowed to complete a visit. As this meant that not all insects could be caught and identified to species, we assigned all visitors into one of 29 primary visitor groups (“lower resolution”; this classification is hereafter used for all analyses unless otherwise stated) and one of 49 higher taxonomic resolution visitor categories. Full details of visitor groups and specimens identified by taxonomists can be found in Supplementary Table S1. In most cases, the lower resolution classification was genus level; but, for example, the “Osmiini” refers to the genera *Osmia*, *Protosmia*, and *Hoplitis* (excluding the more distinctive *Chelostoma* which are grouped separately). For the higher level resolution, classification was split into size categories within genera where possible, all distinguishable by eye (e.g. *Eucera* was divided into *Eucera* large, medium and small), since visitors of different sizes may vary in their pollination effectiveness^45^. While this lead to taxonomically uneven groupings, the same groupings were used consistently for all analysis.

### Data analysis

#### Visitation as a predictor of pollen deposition

Spearman rank correlations were used to test for relationships between plant-visitor interaction strengths for V and both PE and PI (question 1). As these values were proportional, they were arcsine-square-root transformed before analysis. Individual Spearman rank correlations were also used to compare V and PE proportions across visitor groups which had links with four or more plant species (all Coleoptera were combined, resulting in 13 visitor groups).

#### Pollen deposition analysis

To evaluate variation in pollen deposition ability among different visitor groups (question 2) data were analysed both at the community level and the level of individual plant species. Visitor groups with fewer than five recorded visits (*Bombus*, Orthoptera, *Pygopleurus* and *Tropinota*) were excluded from analyses.

Pollen deposition was analysed for each plant species using raw pollen grain counts (i.e. before control values were subtracted). The distribution of pollen counts was both zero-inflated and over-dispersed, so a hurdle approach was used^46, 47^, in which a binomial family GLM was used to model the probability of a non-zero count and then a negative-binomial family model used to predict the number of grains present given a non-zero count. In both models, visitor group was entered as a categorical predictor with control (bagged) flowers as the baseline level to determine which visitors were significantly more likely to deposit pollen onto stigmas than controls and, when pollen was deposited, which visitors deposited significantly more pollen than found on controls. Time of visit and length of visit were included as continuous covariates in both models. Predicted pollen counts were obtained by multiplying the predicted probability of a non-zero count from the binomial model by the expected count from the negative-binomial model. At the community level a Spearman Rank correlation was used to test for a broad relationship between pollen deposition and time of day.

#### Network construction

The data were used to construct three types of bipartite networks^6, 48^:

**1) Visitation (V) network**, using the interaction frequency between visitor groups and plant species. Sample sizes varied slightly among plant species (Table 1), so visit frequency for a plant-visitor group interaction was calculated as a proportion of the total number of visits by all visitor groups; thus interaction bar widths sum to 1 for each plant species, removing bias from variation in sampling effort and facilitating valid comparisons with equivalent PE and PI networks.

**2) Pollinator Effectiveness (PE) network**, using mean SVD values for each visitor group to each plant species. Pollen production (and hence deposition) varied greatly between plant species. If raw counts of pollen grains were used to produce an interaction network the strength of interactions would be heavily weighted by pollen production per plant species. To remove such bias from the PE network, the PE interaction between each visitor group and plant species was calculated as a proportion of the total SVD for that plant species (i.e. total pollen grains deposited across all SVD observations).

**3) Pollinator Importance (PI) network**, combining data from V and PE networks. PI for each interaction was calculated as the product of total visitation frequency and mean PE for that visitor group. Again, biases were accounted for by using PI values for each visitor group/plant species interaction calculated as a proportion of the total PI summed across all visitor groups for that plant, so that all interaction bar widths for each plant species sum to 1.

Network construction was repeated for different versions of the data to assess the consistency of the findings: (1) visitor groups classified at the higher taxonomic level. (2) All interaction links with fewer than 5 visits removed. (3) Data were split into “early” (collected from13^th^ Feb to 21^st^ March) or “late” (collected from 22^nd^ March to 25^th^ April) season, as plant and visitor phenophases varied over the season (six species were sampled throughout the sampling period were classed as “both” early and late) (Table 1). (4) Only visitor groups depositing significantly more pollen compared to control flowers included (identified using the hurdle model detailed above).

#### Comparing visitation networks with PE and PI networks

Interaction networks were analysed using the bipartite package (version 2.05^46^, in R version 3.2.1^49^). Our analyses focused on metrics that best represented community structure and the role of individual species within that structure. Community-level metrics were used to test for large scale differences between networks (question 3).

We used H_2_′ to measure network specialisation as it best represents the level of interaction selectiveness by estimating the deviation of observed interaction frequencies from expected values observed in a null distribution of interactions. H_2_′ is based on weighted links and is therefore robust to variation in sampling effort because its null expectation is unaffected by sampling intensities^50^; it ranges from 0 (extreme generalisation) to 1 (perfect specialisation). **Generality** of visitor species, together with generality of plant species (the latter is also termed ‘vulnerability’ due to its use in food web literature, describing the vulnerability of prey to predation^51^), were used to describe the mean numbers of species a plant or visitor group directly interacts with, weighted to account for sample size. **Interaction evenness** was used as a measure of homogeneity in interaction frequencies, which reaches 1 when the number of interactions between plants and visitor groups is uniformly distributed, and is inversely related to network stability^52^. **Nestedness**, weighted by sample size (weighted NODF), was used as an estimate of linkage structure, which may inform about network dynamics and breakdown patterns, with higher values of nestedness correlated with greater stability in mutualistic networks^53, 54^.

We expect PI networks to be more specialised, with a higher H_2_′, with a lower generalisation and with lower nestedness when interaction links involving visitors with poor pollinator effectiveness scores are reduced in importance. Potential change in network evenness was not predicted, as the changes in species contribution within the network could lead to changes in either direction. To ensure comparisons of network metrics were between the most unbiased estimates, observed network-level metrics were corrected using the means from 1000 null model distributions (produced using the Patefield algorithm, in which the marginal totals are constrained in the randomizations^55^). Standardized z-scores were calculated for each network level metric (z = [observed − null mean]/null ơ) to test for significant difference from the null model distribution^54^.

#### Comparing species/group-level metrics with morphological traits

To identify possible links between morphological traits and species-level network metrics (question 4), the 23 plant species were classified on a scale of 1 to 5 using the judgement of the authors for each of three morphological characteristics: (i) ease of stigma access (ii) size of receptive stigma surface and (iii) pollen grain size (Table 1). The body size and tongue length of each of the 29 visitor groups were classified on a scale of 1 to 5 (Supplementary Table S3a). Full definitions for plant and insect categorisation provided in Supplementary Material S1 and Supplementary Table S2.

We used d′ to assess species/group level specialisation; it measures the exclusivity of interactions that individual species take part in. This is the most biologically informative measure of visitor specialisation in resource choice in a visitation network, and most relevant predictor of specialisation in pollination for a plant in a PI network^56^. As the matrix data are proportional, all values were multiplied by 1000 to allow calculation of this metric. **Species strength**, on the other hand, measures the sum of an individual species’ or groups’ dependencies (relative interaction weights) within a network^15^; this measure of species contribution to the other trophic level is most biologically informative for plants in a visitation network (as resource use of these species by visitors is measured), and for visitors in a PI network (where potential pollination quality is measured). We then used **weighted closeness centrality** to provide a measure of the centrality of individual species/groups within the topography of the network that accounted for sample size, with the highest values indicating generalised keystone species with more linkages to other centrally-located species and lower values indicating marginal species/groups that were less likely to interact with keystone generalists, or species that in turn interacted with keystone generalists^57^. Differences in these species/group-level metrics between V and PI networks depend on changes in individual interactions, making network-level predictions difficult.

Plant species level metrics d′ and weighted closeness centrality from the PI network, and species strength from the V network, were tested for relationships with ratings of stigma access, stigma size and pollen grain size using Spearman rank correlations. Visitor group level metrics d′ and weighted closeness centrality from the V network and species strength from the PI network were assessed in the same way for relationships with visitor body size and tongue length.

## Electronic supplementary material

Supplementary Material Files

Supplementary Table S1

Supplementary Table ﻿S2Supplementary Table S3a

Supplementary Table S3b

Sup﻿plementary Table S4Supplementary Table S5

Supplementary Table S6

Supplementary Table S7

## References

[1] Tylianakis JM, Didham RK, Bascompte J, Wardle DA (2008). Global change and species interactions in terrestrial ecosystems. Ecol. Lett..

[2] Sabatino M, Maceira N, Aizen MA (2010). Direct effects of habitat area on interaction diversity in pollination webs. Ecol. Appl..

[3] Valiente-Banuet A (2014). Beyond species loss: the extinction of ecological interactions in a changing world. Funct. Ecol..

[4] Hicks DM (2016). Food for pollinators: quantifying the nectar and pollen resources of urban flower meadows. PLoS ONE.

[5] Garibaldi LA (2013). Wild pollinators enhance fruit set of crops regardless of honey bee abundance. Science.

[6] Ballantyne G, Baldock KCR, Willmer PG (2015). Constructing more informative plant–pollinator networks: visitation and pollen deposition networks in a heathland plant community. Proc. Roy. Soc. Lond..

[7] Ne’eman G, Jürgens A, Newstrom-Lloyd L, Potts SG, Dafni A (2010). A framework for comparing pollinator performance: effectiveness and efficiency. Biol. Rev. Camb. Philos. Soc..

[8] Vázquez DP, Morris WF, Jordano P (2005). Interaction frequency as a surrogate for the total effect of animal mutualists on plants. Ecol. Lett..

[9] Vázquez DP, Lomáscolo SB, Maldonado MB, Chacoff NP, Dorado J (2012). The strength of plant-pollinator interactions. Ecology.

[10] Arceo-Gómez G, Ashman TL (2014). Patterns of pollen quantity and quality limitation of pre-zygotic reproduction in *Mimulus guttatus* vary with co-flowering community context. Oikos.

[11] Gibson RH, Knott B, Eberlein T, Memmott J (2011). Sampling method influences the structure of plant–pollinator networks. Oikos.

[12] Ollerton J, Rech AR, Waser NM, Price MV (2015). Using the literature to test pollination syndromes - some methodological cautions. J Pollinat. Ecol..

[13] Kaiser-Bunbury CN, Blüthgen N (2015). Integrating network ecology with applied conservation: a synthesis and guide to implementation. AoB Plants.

[14] Brosi BJ, Briggs HM (2013). Single pollinator species losses reduce floral fidelity and plant reproductive function. Proc. Natl Acad. Sci. USA.

[15] Bascompte J, Jordano P, Olesen JM (2006). Asymmetric coevolutionary networks facilitate biodiversity maintenance. Science.

[16] Chamberlain SA (2014). Traits and phylogenetic history contribute to network structure across Canadian plant–pollinator communities. Oecologia.

[17] Willmer, P. G., Cunnold, H. & Ballantyne, G. Insights from measuring pollen deposition – quantifying the pre-eminence of bees as flower visitors and effective pollinators. *Arthropod Plant Interact* (in press).

[18] Johnson SD, Dafni A (1998). Response of bee-flies to the shape and pattern of model flowers: Implications for floral evolution in a Mediterranean herb. Funct. Ecol..

[19] Patchett, R., Ballantyne, G. & Willmer, P. G. Estimating pollinator performance of visitors to the self-incompatible crop-plant Brassica rapa by single visit deposition and pollen germination: a comparison of methods. *J Pollinat Ecol* (in press).

[20] Cunnold, H. & Willmer, P. G. Comparisons between visitation, pollinator importance and pollen transport networks in an urban garden. (in prep).

[21] Jauker F, Wolters V (2008). Hover flies are efficient pollinators of oilseed rape. Oecologia.

[22] Stavert, J.R. *et al*. Hairiness: the missing link between pollinators and pollination. *PeerJ* **4**, e2779 (2016). 10.7717/peerj.2779 PMC5180583 28028464

[23] Chacoff NP, Aizen MA, Aschero V (2008). Proximity to forest edge does not affect crop production despite pollen limitation. Proc. Roy. Soc. Lond..

[24] Frier SD, Somers CM, Sheffield CS (2016). Comparing the performance of native and managed pollinators of Haskap (*Lonicera caerulea*: Caprifoliaceae), an emerging fruit crop. Agric. Ecosyst. Environ..

[25] Willmer, P. G. *Pollination and Floral Ecology* (Princeton University Press, 2011).

[26] Popic TJ, Wardle GM, Davila YC (2013). Flower-visitor networks only partially predict the function of pollen transport by bees. Austral Ecol..

[27] Vizentin-Bugoni J, Maruyama PK, Sazima M (2014). Processes entangling interactions in communities: forbidden links are more important than abundance in a hummingbird-plant network. Proc. Roy. Soc. Lond..

[28] Watts S, Ovalle DH, Herrera MM, Ollerton J (2012). Pollinator effectiveness of native and non-native flower visitors to an apparently generalist Andean shrub, *Duranta mandonii* (Verbenaceae). Plant Species Biol..

[29] Roque BB, Pena RS, Salas A, Koptur S (2016). Butterflies visit more frequently, but bees are better pollinators: the importance of mouthpart dimensions in effective pollen removal and deposition. AoB Plants.

[30] Ali M, Saeed S, Sajjad A (2016). Pollen deposition is more important than species richness for seed set in luffa gourd. Neotrop. Entomol..

[31] Frick WF, Price RD, Heady PA, Kay KM (2013). Insectivorous bat pollinates columnar cactus more effectively per visit than specialized nectar bat. Am. Nat..

[32] Mayfield MM, Waser NM, Price MV (2001). Exploring the ‘most effective pollinator principle’ with complex flowers: bumblebees and Ipomopsis aggregate. Ann. Bot..

[33] Junker RR, Blüthgen N, Keller A (2015). Functional and phylogenetic diversity of plant communities differently affect the structure of flower-visitor interactions and reveal convergences in floral traits. Evol. Ecol..

[34] Baldock KCR, Memmott J, Ruiz-Guajardo JC, Roze D, Stone GN (2011). Daily temporal structure in African savanna flower visitation networks and consequences for network sampling. Ecology.

[35] Dattilo W (2014). Individual-based ant-plant networks: Diurnal-nocturnal structure and species-area relationship. PLoS ONE.

[36] Ramsey M, Vaughton G (2000). Pollen quality limits seed set in *Burchardia umbellata* (Colchicaceae). Am. J. Bot..

[37] Greenleaf SS, Williams NM, Winfree R, Kremen C (2007). Bee foraging ranges and their relationship to body size. Oecologia.

[38] Morris WF, Vázquez DP, Chacoff NP (2010). Benefit and cost curves for typical pollination mutualisms. Ecology.

[39] Aizen MA, Morales CL, Diego PV (2014). When mutualism goes bad: density-dependent impacts of introduced bees on plant reproduction. New Phytol..

[40] Chacoff NP, García D, Obeso JR (2008). Effects of pollen quality and quantity on pollen limitation in Crataegus monogyna (Rosaceae) in NW Spain. Flora.

[41] Encinas-Viso F, Revilla TA, Etienne RS (2012). Phenology drives mutualistic network structure and diversity. Ecol. Lett..

[42] Rasmussen C, Dupont YL, Mosbacher JB, Trøjelsgaard K, Olesen JM (2013). Strong impact of temporal resolution on the structure of an ecological network. PLoS ONE.

[43] Alarcón R (2010). Congruence between visitation and pollen-transport networks in a California plant-pollinator community. Oikos.

[44] King C, Ballantyne G, Willmer PG (2013). Why flower visitation is a poor proxy for pollination: Measuring single-visit pollen deposition, with implications for pollination networks and conservation. Methods Ecol. Evol..

[45] Willmer PG, Finlayson K (2014). Big bees do a better job: Intraspecific size variation influences pollination effectiveness. J. Pollinat. Ecol..

[46] Cameron, A. C. & Trivedi, P. K. *Regression analysis of count data*. Cambridge University Press, Cambridge (1998).

[47] Zeileis A, Kleiber C, Jackman S (2008). Regression models for count data in R. Journal of Statistical Software.

[48] Dormann CF, Fruend J, Bluethgen N, Gruber B (2009). Indices, graphs and null models: analyzing bipartite ecological networks. TOECOLJ.

[49] R Core Team. R: a language and environment for statistical computing. Vienna, Austria: R Foundation for Statistical Computing. http://www.R-project.org/ (2014).

[50] Blüthgen N, Fründ J, Vázquez DP, Menzel F (2008). What do interaction network metrics tell us about specialization and biological traits?. Ecology.

[51] Bersier LF, Banašek-Richter C, Cattin MF (2002). Quantitative descriptors of food-web matrices. Ecology.

[52] Rooney N, McCann KS (2012). Integrating food web diversity, structure and stability. Trends Ecol. Evol..

[53] Allesina S, Tang S (2012). Stability criteria for complex ecosystems. Nature.

[54] Almeida-Neto M, Guimarães P, Guimarães PR, Loyola RD, Ulrich W (2008). A consistent metric for nestedness analysis in ecological systems: reconciling concept and measurement. Oikos..

[55] Patefield WM (1981). Algorithm AS159. An efficient method of generating r x c tables with given row and column totals. Applied Statistics.

[56] Blüthgen N, Menzel F, Blüthgen N (2006). Measuring specialization in species interaction networks. BMC Ecology.

[57] Martín González AM, Dalsgaard B, Olesen JM (2010). Centrality measures and the importance of generalist species in pollination networks. Ecol. Complexity.

