# Author Correction: Pollinator importance networks illustrate the crucial value of bees in a highly speciose plant community

**DOI:** 10.1038/s41598-020-78538-1

**Published:** 2020-12-08

**Authors:** Gavin Ballantyne, Katherine C. R. Baldock, Luke Rendell, P. G. Willmer

**Affiliations:** 1 University of St Andrews, School of Biological Sciences, St Andrews, KY16 9TH UK; 2 Edinburgh Napier University, School of Applied Sciences, Edinburgh, EH11 4BN UK; 3 University of Bristol, School of Biological Sciences, Bristol, BS8 1TQ UK; 4 University of Bristol, Cabot Institute, Bristol, BS8 1UJ UK

Correction to: *Scientific Reports* 10.1038/s41598-017-08798-x, published online 21 August 2017

This Article contains errors in the HTML where the labelling of Electronic supplementary material is incorrect.

“Supplementary Table S2Supplementary Table S3a”

should read:

“Supplementary Table S3a”,

And “Supplementary Table S4Supplementary Table S5”

should read:

“Supplementary Table S5”

Additionally, the Supplementary Table S3a file that accompanies this Article contains errors.

The value in cell 26E is omitted and should read: “0 (1)”.

The value in cell 19Y, "65.75 ± 31.34 (31)", should read: “65.75 ± 31.34 (8)”.

A row of data on line 34 is omitted and should show the mean control counts for each species.

The corrected Supplementary Table S3a file is linked to this correction notice.

## Supplementary Information

Supplementary Table 3a.

