# Supplementary material for: Pollinator importance networks illustrate the crucial value of bees in a highly speciose plant community
Source: Sci Rep. 2017 Aug 21;7:8389. doi: 10.1038/s41598-017-08798-x (PMC5566368; doi:10.1038/s41598-017-08798-x)
Supplement: Supplementary file 1 — Supplementary Material Files [file 41598_2017_8798_MOESM1_ESM.doc]

**Pollinator importance networks illustrate the crucial value of bees in a highly speciose plant community**

Gavin Ballantyne, Katherine C R Baldock, Luke Rendell, P G Willmer

**Supplementary Material Legends**

**Supplementary Figure S1:** Observed and predicted (+/- 95% bootstrap CI) pollen deposition counts by visitor for each plant species.

**Supplementary Table S1:** Taxonomy of visitor groups, from a sub sample of collected specimens.

**Supplementary Table S2:** Definitions for plant and visitor morphological trait categories.

**Supplementary Table S3a:** Mean pollen deposition of primary visitor groups to plant species, with standard errors and sample sizes. Mean values accounted for pollen already present on stigmas by subtracting control means for that plant species before being calculated. Visitor group body size and tongue length categories are also shown.

**Supplementary Table S3b:** Mean pollen deposition of visitor groups to plant species at a finer degree of taxonomic resolution, with standard errors and sample sizes.

**Supplementary Table S4:** Individual Spearman rank correlations between proportions of V and PE across the visitor groups where interactions with four or more plant species occurred (Coleoptera were also analysed together, resulting in 13 visitor groups). Significant or marginally significant p values in bold.

**Supplementary Table S5:** Output from hurdle model of pollen deposition at the species level, with output for pollen presence and pollen deposition for each plant species. All visitor groups were included as factors and time of day and length of visit were included as covariates.

**Supplementary Table S6:** Community level network metrics for the standard dataset, dataset with small sample sizes removed, dataset with visitor groups split to a finer taxonomic resolution, datasets for early and late season flowering, and dataset with only significantly effective visitors identified by hurdle model included. Observed network metrics, mean and standard deviation of null model predictions (1000 null models) and metrics corrected by null model predictions are shown for each network. Number of interactions are controlled for in the null model, so are not predicted. Z-scores ([observed - null mean]/null standard deviation) shown in bold where observed values significantly different from null models (by two or more standard deviations; p<0.001).

**Supplementary Table S7:** Species level network metrics d’, species strength and weighted closeness centrality for plants and visitor groups in the V, PE and PI networks.


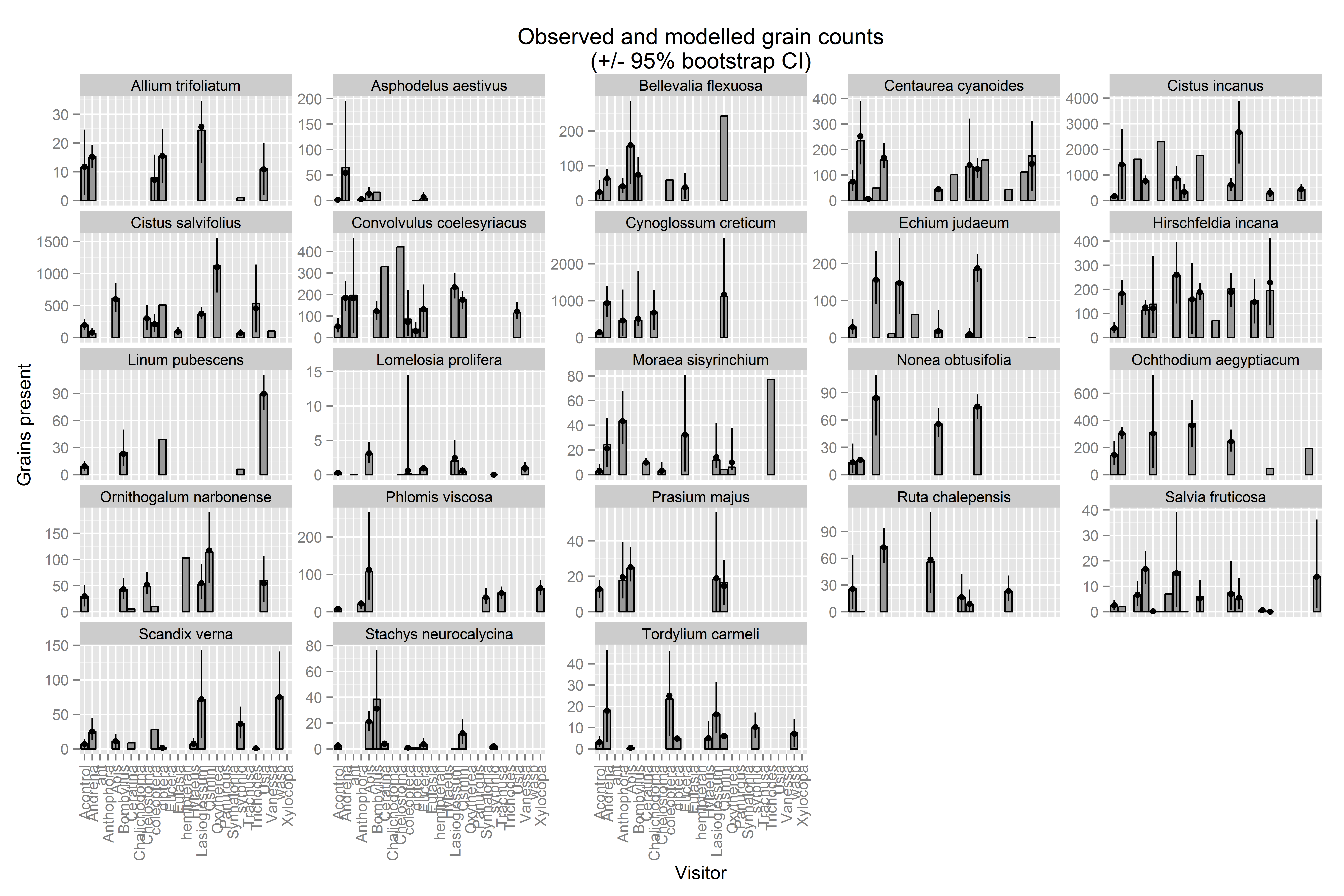
 **Supplementary Figure S1:**

**Supplementary Material S1: Definitions for plant and visitor morphological trait categories.**

To identify possible links between morphological traits and species-level network metrics (question 4), the 23 plant species were classified on a scale of 1 to 5 using the judgement of the authors for each of three morphological characteristics: (i) ease of stigma access, where 1 corresponds to species with very restricted access such as *Phlomis viscosa* (visitors have to force open the robust upper petal to make contact with the stigma), and 5 corresponds to species such as *Cistus incanus* (stigma fully exposed to visitors of any size or shape); (ii) size of receptive stigma surface (surface of whole stigma, or combined surface where multiple stigmas are present in a single flower) and (iii) pollen grain size (full definitions provided in Supplementary Table S2).

The body size and tongue length of each of the 29 visitor groups were classified on a scale of 1 to 5 (listed in Supplementary Table S3a), where a visitor with body size and tongue length of 1 describes an ant or very small orthopteran nymph and a body size and tongue length of 5 describe large long-tongued bees like *Synhalonia* (full definitions provided in Supplementary Table S2). *Bombus terrestris* was excluded due to small sample sizes.

**Supplementary Table S2:**

| **Morphological trait definitions** | |
| --- | --- |
| Criteria | Definition |
| **Stigma access** | |
| 1 | stigma not accessible unless flower manipulated to expose stigma |
| 2 | stigma recess into a hood or covering of hairs within the corolla |
| 3 | visitor required to insert mouth parts into an open corolla to contact stigma |
| 4 | Stigma fully exposed to small visitors, larger visitors may walk over the stigma without contact |
| 5 | Stigma fully exposed to visitors of all sizes and shapes |
| **Stigma size (total receptive surface area of stigma)** | |
| 1 | >0.1mm |
| 2 | 0.1-0.4mm |
| 3 | 0.5-0.8mm |
| 4 | 0.9-1.2mm |
| 5 | >1.2mm |
| **Pollen size** | |
| 1 | <10μm |
| 2 | 10-20μm |
| 3 | 20-30μm |
| 4 | 35-50μm |
| 5 | 50μm |
| **Visitor size** | |
| 1 | <5mm |
| 2 | 5-10mm |
| 3 | 10-15mm |
| 4 | 15-20mm |
| 5 | >20mm |
| **Visitor tongue length** | |
| 1 | <1mm |
| 2 | 1-3mm |
| 3 | 3-6mm |
| 4 | 6-10mm |
| 5 | >10mm |

**Supplementary Table S4:**

| **Visitor Group** | **Spearman rho** | **p value** |
| --- | --- | --- |
| *Andrena* | 0.496 | **0.051** |
| *Anthophora* | 0.227 | 0.503 |
| *Apis* | 0.332 | 0.246 |
| *Bombylius* | 0.155 | 0.649 |
| *Ceratina* | 0.431 | 0.286 |
| *Chelostoma* | 0.050 | 0.912 |
| Coleoptera | -0.131 | 0.583 |
| *Eucera* | 0.315 | 0.318 |
| *Lasioglossum* | 0.069 | 0.793 |
| Osmini | 0.196 | 0.502 |
| other Diptera | 0.384 | 0.217 |
| syrphids | 0.782 | **0.002** |
| *Usia* | 0.486 | 0.356 |
